# Late Atrio-ventricular Block After Arterial Switch for D-transposition of the Great Vessels With Intact Interventricular Septum

**DOI:** 10.4021/cr69w

**Published:** 2011-09-20

**Authors:** Guillaume Caudron, Sebastien Hascoet, Yves Dulac, Philippe Maury

**Affiliations:** a Department of Cardiology, University Hospital Rangueil, Toulouse, France; b Department of Cardiology, Children University Hospital, Toulouse, France

**Keywords:** Transposition of the great arteries, Arterial switch, Atrio-ventricular block, Atrial septal defect, Ventricular septal defect

## Abstract

Arterial switch operation for transposition of the great arteries without ventricular septal defect usually does not lead to atrio-ventricular conduction disturbances. We discuss the case of a young boy presenting with first and second degree supra hisian atrio-ventricular block late after switch operation.

## Introduction

Arterial switch operation for transposition of the great arteries without ventricular septal defect usually does not lead to atrio-ventricular (AV) conduction disturbances. We discuss the case of a young boy presenting with first and second degree supra hisian AV block late after switch operation.

## Case Report

A 15-year-old boy was referred for repetitive near-syncopes. He presented at birth with a D-transposition of the great arteries associated to an ostium secundum atrial septal defect and intact interventricular septum. An arterial switch operation had been performed at day 2, together with closure of the atrial septal defect by suture. One year later, he underwent a surgical enlargement of a supravalvular pulmonary artery stenosis secondary to the arterial switch operation. First degree AV block and complete right bundle branch block were present post-operatively (first available ECG in this patient). Until 15-year-old, he remained asymptomatic with stable yearly echocardiographical follow-up, but with a progressively increasing PR interval.

**Figure 1:**
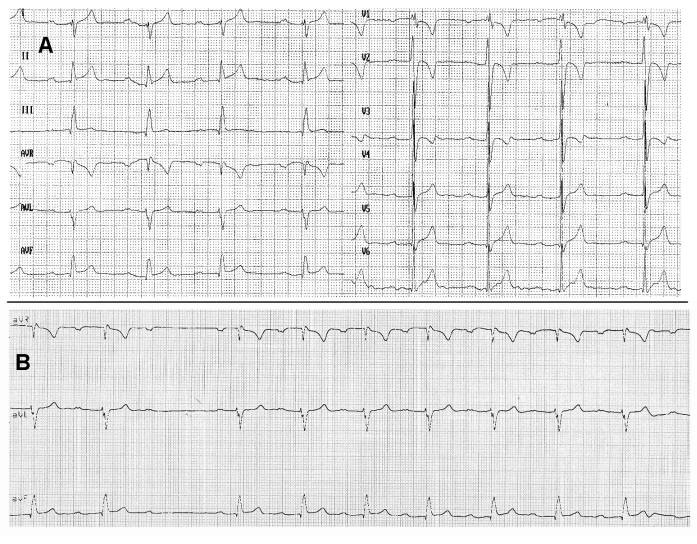
(A) Baseline ECG of the patient showing sinus rhythm with first degree AV block, right bundle branch block and right axis (25 mm/sec). Tall morphology of the patient may explain the right inferior QRS axis, although left posterior fascicular block can not be ruled out (even if unlikely due to the normal HV interval and lack of surgical injury on the inferior ventricular septum). Right bundle branch block may be the consequence of the surgery of the supravalvular pulmonary artery stenosis as realized at one year of life or of the right ventricular overload because of the pulmonary artery stenosis. Alternatively intraventricular conductions disturbances have also been described following surgical closure of atrial septal defect (see text for explanations). (B) Baseline ECG strip (VR, VL, VF leads) revealing spontaneous type 1 second degree AV block (25 mm/sec)

He was then referred for repetitive episodes of near-syncopes associated with perceptions of decreased heart rate and cardiac pauses. Baseline ECG registered first degree (PR 380 milliseconds) or type 1 second degree AV block with complete right bundle branch block and right inferior axis (Fig. 1). Repeated 24 hours ambulatory recordings revealed only nocturnal Mobitz 1 second degree AV block, but no symptoms happened during these recordings, while AV conduction normalizes during treadmill.

**Figure 2:**
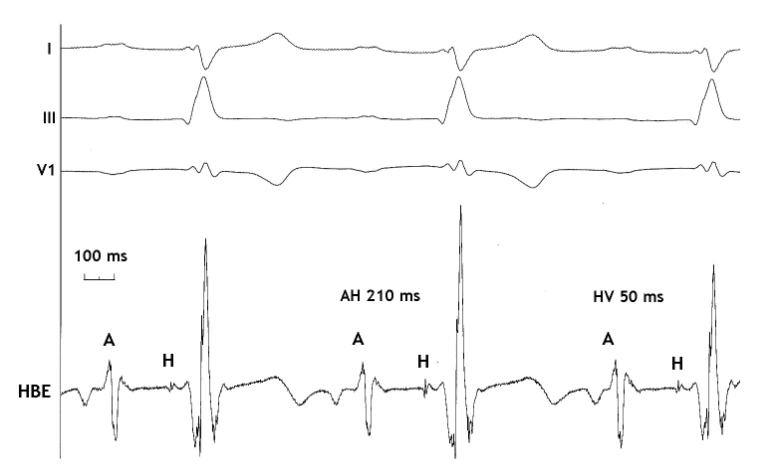
Intra-cardiac recording showing the long AH interval (210 milliseconds) and normal HV interval (50 milliseconds). I, III and V1: surface electrocardiogram leads. HBE: His bundle recording.

A normal cardiac CT scan eliminated coronary artery abnormalities. Electrophysiological investigation found prolonged AH interval (210 milliseconds) during phases of first degree AV block (Fig. 2), a supra Hisian Wenckebach phenomenon at an atrial pacing rate of 650 milliseconds together with a prolonged AV node refractory period (520 milliseconds). H potential and HV interval (50 milliseconds) were normal, as were sinus node function electrophysiological tests.

According to the current guidelines, a single lead-dual chamber permanent pacemaker (VDD) was then implanted for these conduction disturbances suspected to be linked to the symptoms, even if no ECG documentation was available at the time of faints. The pacemaker was initially programmed in slow VVI backup mode with hysteresis. However, the patient came back a few weeks later for repeated faints related to paroxysmal sinus bradycardia due to vaso-vagal attacks, which spontaneously recover without change in pace-maker programming. Over a follow-up of 12 months the patient remained completely asymptomatic and AV conduction unchanged.

## Discussion

Current standard surgical treatment for transposition of the great arteries is represented by the arterial switch operation, realized in the first days of life. Aorta and pulmonary artery roots are cut off and reimplanted on their normal corresponding ventricle, with further reimplantation of the coronary arteries. This surgery replaces atrial switch surgery (Senning and Mustard procedures) for twenty years, which exposed to frequent serious late arrhythmic complications. However bradycardias have also been reported after arterial switch operation: 2nd degree (0.6%) and complete AV block (2%) in a series of 624 patients, with the main risk factor being the existence of an associated ventricular septal defect (3.9% complete AV block with ventricular septal defect versus 1% without) [1]. Since ventricular septal defect is associated to transposition of the great arteries in about 25% and since the rate of complete AV block after surgical closure of isolated perimembranous ventricular septal defect is about 1%, it is therefore licit to assume that the risk of post-operative AV block after arterial switch associated with ventricular septal defect closure is at least similar.

Rhodes and colleagues also demonstrated on 390 patients over a follow-up of eight years, that AV block could be a complication of arterial switch operation: 3% of complete AV block, 1% of second degree AV block and 3% of first degree AV block in case of associated ventricular septal defect, compared to only 2% of first degree AV block and no second or complete AV block when the ventricular septum was intact [2]. Of 158 of the patients without ventricular septal defect investigated one year after the surgery, AH and HV intervals were found prolonged in respectively 5% and 3% of cases [2]. In the series of Di Donato, 1 and 3 of 59 patients treated by arterial switch for transposition of the great arteries with ventricular septal defect presented with postoperative first and third degree AV block respectively, and no further AV block happened over a follow-up of 20 months [3]. Conversely, no AV block was noted by Vetter at one year in 20 patients with arterial switch operation for transposition of the great arteries without ventricular septal defect, and electrophysiological investigations in these patients did not reveal AV node conduction abnormalities [4].

However, there are also alternative explanations for the occurrence of AV block in our patient. He also presented with an ostium secondum atrial septal defect, which was surgically closed. It is known that supra-hisian AV block may complicate the natural or post-operative evolution of ostium secundum atrial septal defect. Preoperative Holter recordings and electrophysiological studies in 40 children with atrial septal defect revealed 15% of first degree AV block and 18% displayed altered AV node conduction [5]. Mutations in NKX2.5 gene could be involved in such cases. Surgical closure of atrial septal defect has also been incriminated in AV block: Wall and colleagues found 6 cases of intra-ventricular conduction disturbances and 2 first degree AV block over a 30 years follow-up of 14 patients after surgical closure of ostium secondum atrial septal defect during childhood [6]. In fact, arterial switch operation very frequently needs associated surgical atrial septal defect closure due to an associated atrial septal defect or to the Rashkind septostomy. This simple fact alone may explain the occurrence of AV block in the setting of arterial switch operation. Other operations that require the Rashkind procedure preoperatively such as the Senning or Mustard procedures also have the potential for postoperative AV block, but role of septostomy closure in postoperative conduction disturbances can not be determined since these surgeries also carry some intrinsic risk for AV block.

### Conclusions

Arterial switch operation for transposition of the great arteries may cause late AV block even in the absence of closure of associated ventricular septal defect. Associated atrial septal defect or closure of such atrial septal defect or of Rashkind septostomy may also explain some of these AV blocks.
